# Repair of tracheal epithelium by basal cells after chlorine-induced injury

**DOI:** 10.1186/1465-9921-13-107

**Published:** 2012-11-22

**Authors:** Sadiatu Musah, Jing Chen, Gary W Hoyle

**Affiliations:** 1 Department of Environmental and Occupational Health Sciences, School of Public Health and Information Sciences, University of Louisville, 701 HSC-A, 319 Abraham Flexner Way, Louisville, KY, 40202, USA

**Keywords:** Acute lung injury, Tracheobronchial epithelium, Re-epithelialization

## Abstract

**Background:**

Chlorine is a widely used toxic compound that is considered a chemical threat agent. Chlorine inhalation injures airway epithelial cells, leading to pulmonary abnormalities. Efficient repair of injured epithelium is necessary to restore normal lung structure and function. The objective of the current study was to characterize repair of the tracheal epithelium after acute chlorine injury.

**Methods:**

C57BL/6 mice were exposed to chlorine and injected with 5-ethynyl-2^′^-deoxyuridine (EdU) to label proliferating cells prior to sacrifice and collection of tracheas on days 2, 4, 7, and 10 after exposure. Airway repair and restoration of a differentiated epithelium were examined by co-localization of EdU labeling with markers for the three major tracheal epithelial cell types [keratin 5 (K5) and keratin 14 (K14) for basal cells, Clara cell secretory protein (CCSP) for Clara cells, and acetylated tubulin (AcTub) for ciliated cells]. Morphometric analysis was used to measure proliferation and restoration of a pseudostratified epithelium.

**Results:**

Epithelial repair was fastest and most extensive in proximal trachea compared with middle and distal trachea. In unexposed mice, cell proliferation was minimal, all basal cells expressed K5, and K14-expressing basal cells were absent from most sections. Chlorine exposure resulted in the sloughing of Clara and ciliated cells from the tracheal epithelium. Two to four days after chlorine exposure, cell proliferation occurred in K5- and K14-expressing basal cells, and the number of K14 cells was dramatically increased. In the period of peak cell proliferation, few if any ciliated or Clara cells were detected in repairing trachea. Expression of ciliated and Clara cell markers was detected at later times (days 7–10), but cell proliferation was not detected in areas in which these differentiated markers were re-expressed. Fibrotic lesions were observed at days 7–10 primarily in distal trachea.

**Conclusion:**

The data are consistent with a model where surviving basal cells function as progenitor cells to repopulate the tracheal epithelium after chlorine injury. In areas with few remaining basal cells, repair is inefficient, leading to airway fibrosis. These studies establish a model for understanding regenerative processes in the respiratory epithelium useful for testing therapies for airway injury.

## Introduction

Chlorine is a widely used industrial compound and is considered a chemical threat agent that could be intentionally released in an attack on the U.S. populace [1]. Chlorine inhalation injures epithelial cells of both the upper and lower airways leading to acute effects of pulmonary edema, pneumonitis, and pulmonary function abnormalities [2,3]. High-level chlorine exposure results in sloughing of the pseudostratified airway epithelium of the proximal airways composed primarily of secretory, ciliated, and basal cells [4-6]. Repair of the airways after injury involves the coordinated action of local progenitor cells and stem cells to restore the integrity of the epithelium [7-9]. Understanding these processes following chlorine lung injury may suggest strategies for treating injury or accelerating epithelial repair.

In human airways, a pseudostratified epithelium containing basal epithelial cells is present in the trachea, bronchi, and multiple generations of bronchioles down to fairly small airways. In contrast, mice have a pseudostratified epithelium in the trachea, but this transitions rapidly in the bronchi to a simple epithelium lacking basal cells. For these reasons, repair processes in the mouse trachea are likely to be most relevant to those occurring in human airways [10]. In mice, basal cells function as progenitor cells to repair tracheal and bronchial epithelium, whereas Clara cells are progenitor cells in bronchiolar epithelium [11-13]. Although the identity of tissue-specific stem cells in the airways is not completely established, current evidence suggests that subsets of basal cells and Clara cells function as stem cells for repair and long-term maintenance of the tracheobronchial and bronchiolar epithelium, respectively [11,14-17]. In the mouse tracheobronchial epithelium, most basal cells express the cytoskeletal protein keratin 5 (K5) whereas only a subset of these cells express keratin 14 (K14) during steady state [9-11]. Lineage-tracing studies show that both K5- and K14-expressing basal cells are capable of extensive self-renewal and differentiation into ciliated and Clara cells [12,18]. Because efficient repair of injured epithelial cells is necessary for restoration of normal lung structure and function, we sought to characterize repair of the tracheal epithelium in mice after acute chlorine injury. Here we report that after chlorine-related tracheal injury, epithelial repair occurs faster in the proximal trachea than in distal trachea/mainstem bronchus with basal cells initiating repair and serving as progenitor cells for the restoration of the tracheal epithelium.

## Materials and methods

### Animals

Experiments involving animals were approved by the University of Louisville Institutional Animal Care and Use Committee and were conducted in accordance with the Institute of Laboratory Animal Resources *Guide for the Care and Use of Laboratory Animals*[19]. Male C57BL/6 mice were purchased from the Jackson Laboratory and randomly assigned to chlorine exposed or unexposed groups. Mice at 9–10 weeks of age were exposed to a target dose of 240 ppm-hr (a concentration of 240 ppm for 1 hr) chlorine in a whole body exposure chamber [4]; deviation between target and actual doses averaged 1.25%. For time-course experiments, mice were injected intraperitoneally with 10 mg/kg of 5-ethynyl-2^′^-deoxyuridine (EdU) [20] from Life Technologies (Grand Island, NY) 17 hrs prior to euthanasia at different times after exposure (days 2, 4, 7, or 10) to label proliferating cells. Mice were euthanized for collection of tracheal tissue by injection with tribromoethanol (375 mg/kg intraperitoneally) followed by exsanguination. Tracheal tissues were collected, fixed in 10% neutral buffered formalin overnight and divided into 3 equal pieces along the proximal-distal axis (designated proximal, middle, and distal) before embedding in paraffin.

### Histology, EdU detection, and immunofluorescence

For histological evaluation of tracheal structure, hematoxylin and eosin staining was performed on paraffin-embedded tracheas. To label proliferating cells in tracheal sections, EdU was detected using the Click-It EdU kit (Life Technologies, Grand Island, NY) according to the manufacturer’s protocol. For dual EdU and immunofluorescence staining, EdU staining was performed first, followed by immunostaining. Briefly, sections were deparaffinized and rehydrated, and antigen retrieval was performed with 10 mM sodium citrate, pH 6.0, containing 0.05% Tween-20 at 95°C for 30 min where necessary (for K5 and K14). This was followed by EdU detection and incubation in blocking solution (1% bovine serum albumin, 5% normal goat or donkey serum, and 0.3% Triton X-100) for 30 min at room temperature. After washing, slides were incubated with primary antibodies for 1 hr at room temperature. The following primary antibodies and dilutions were used: rabbit anti-K5 from Covance (Princeton, NJ) (1: 1000; catalog # PRB-160P); mouse anti-K14 from Thermo Scientific (Fremont, CA) (1: 1000; catalog # MS-115-P1); mouse anti-acetylated tubulin (AcTub) from Sigma-Aldrich (St Louis, MO) (1: 20,000; catalog # T7451); and goat anti-Clara cell secretory protein (CCSP), kindly provided by Dr. Gurmukh Singh (VA Medical Center, Pittsburgh, PA) (1: 1000). After washing, slides were incubated with secondary antibodies for 1 hr at room temperature. The following antibodies (Life Technologies, Grand Island, NY) were used at a dilution of 1:500: Alexa Fluor 594 donkey anti-rabbit IgG for K5 immunofluorescence, Alexa Fluor 594 goat anti-mouse IgG for K14, Alexa Fluor 594 donkey anti-mouse IgG for AcTub, and Alexa Fluor 594 donkey anti-goat IgG for CCSP immunofluorescence. Sections were washed, coverslipped with Prolong Gold antifade reagent with DAPI (Life Sciences), and viewed by epifluorescence.

### Trichrome staining and α-smooth muscle actin immunohistochemistry

Trichrome staining was performed using the Accustain® Trichrome Stain (Masson) kit (Sigma-Aldrich, St. Louis, MO) according to the manufacture’s protocol. For immunohistochemistry, sections were deparaffinized and rehydrated, and endogenous peroxidases were inactivated with methanol containing 0.3% hydrogen peroxide for 30 min. This was followed by incubation in blocking solution (3% bovine serum albumin, 5% normal goat serum, and 0.3% Triton X-100) for 30 min at room temperature. Slides were washed thereafter and incubated with α-smooth muscle actin antibody (Sigma Aldrich, St Louis, MO) (1: 5,000; catalog # A5228) for 1 hr at room temperature. After washing, slides were incubated with biotinylated goat anti-mouse IgG (Jackson ImmunoResearch) (1: 3,500) at room temperature for 1 hr. Sections were washed and incubated with streptavidin-conjugated horseradish peroxidase (Jackson ImmunoResearch) (1: 2,000) for 1 hr at room temperature. After washing, slides were developed by incubation in 50 mM Tris–HCl, pH 7.6, containing 0.006% hydrogen peroxide and 200 μg/ml diaminobenzidine for 15 min in the dark. Sections were washed, counterstained with Gill’s hematoxylin (Sigma Aldrich), dehydrated, and coverslipped with PerMount.

### Morphometry

Cross sections were cut at random depths of proximal, middle, and distal trachea for sampling of the trachea along the proximal-distal axis. Digital images encompassing the full circular profile of tracheal cross sections were captured and analyzed. Morphometric analysis [21,22] was performed using point/intercept counting methods and Image J software [23]. Re-epithelialization of the trachea was assessed by scoring the epithelium overlying intercepts of the basement membrane as normal, reparative, squamous, or denuded as follows: 1) normal – columnar or cuboidal pseudostratified epithelium containing ciliated cells; 2) squamous – thin, flattened epithelium; 3) reparative – cuboidal, often pluristratified epithelium lacking ciliated cells; 4) denuded – no epithelium covering the basement membrane. This information was used to derive the percentage of basement membrane surface area covered by the different epithelial structures. For evaluation of epithelial immunostaining, the volume of structures of interest (EdU, K5, K14, CCSP, or AcTub labeling of airway epithelial cells) was measured by point counting and normalized to basement membrane surface area measured by intercept counting. The volume of structure of interest (i) relative to basement membrane surface area was expressed as V_S_ (i,bm) and calculated using the equation V_S_ (i,bm) = 2(P_i_)(k)/π(I_bm_), where P_i_ is the number of points overlying the structure of interest, k is the line length per test point, and I_bm_ is the number of intersections of lines with the basement membrane [21,22]. Staining of interest was assessed in viable tracheal epithelium; staining in subepithelial tissue and in detached epithelial cells was not included in the analysis. For K5 and K14, the volume of K5- or K14-expressing cells was measured by counting points that fell anywhere within a labeled cell, including unlabeled nuclei surrounded by cytoplasmic staining. For CCSP and AcTub, the volume of stained material was measured by counting only points that fell on CCSP or AcTub staining. The volume fraction (V_v_) of EdU-stained nuclei was calculated as points that fell on EdU-labeled nuclei within the epithelium divided by total points that fell on nuclei within the epithelium.

### Statistical analysis

Data are presented as group means ± standard deviation of the mean (SD). Statistical analysis was performed using Prism 4.0a (GraphPad; La Jolla, CA). Group means were compared between chlorine-exposed and unexposed animals using one-way analysis of variance (ANOVA) with Dunnett’s multiple comparison test. Effects of chlorine exposure among groups (e.g. proximal, middle, and distal trachea) were analyzed by one-way ANOVA with Bonferroni’s multiple comparison test. Differences were considered statistically significant at p < 0.05.

## Results

**Figure 1:**
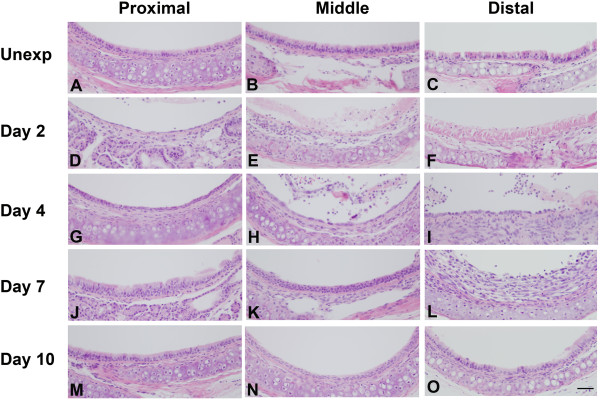
**Histology of tracheal epithelium in chlorine-exposed mice.** Tracheas were collected for histological analysis from chlorine-exposed mice 2, 4, 7, and 10 days after exposure or from unexposed mice (Unexp). Scale bar in Panel O represents 40 μm for all panels.

**Figure 2:**
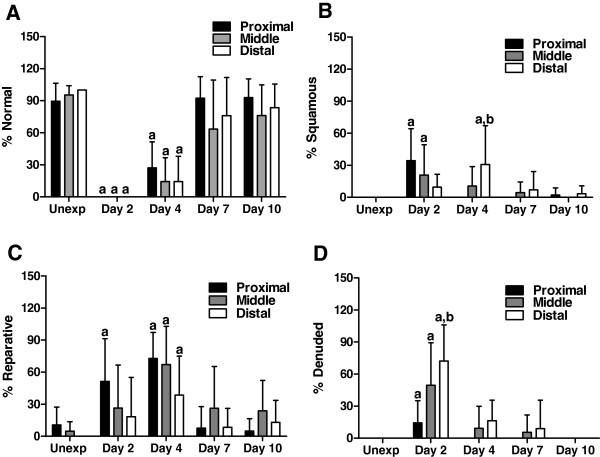
**Morphometric analysis of tracheal epithelium in chlorine-exposed mice.** Tracheal epithelium was evaluated in H&E-stained sections as described in Materials and Methods. The graphs depict the percentage of basement membrane surface area covered by normal (**A**), squamous (**B**), reparative (**C**), or denuded (**D**) epithelium in proximal, middle, and distal trachea. a, p < 0.05 vs. unexposed; b, p < 0.05 vs. proximal.

**Figure 3:**
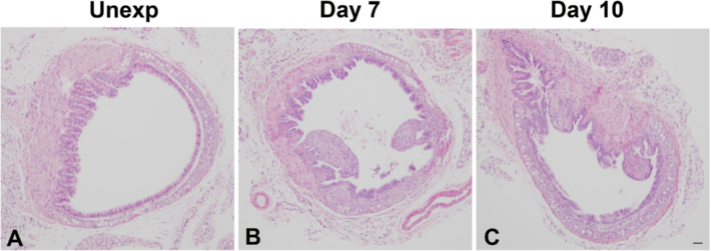
**Airway fibrosis in chlorine-exposed mice.** Tracheas were collected for histological analysis from chlorine-exposed mice 7 and 10 days after exposure or from unexposed mice. Note fibroproliferative lesions on days 7 and 10. Scale bar in Panel **C** represents 50 μm for all panels.

**Figure 4:**
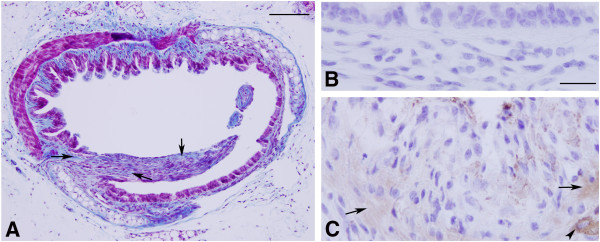
**Trichrome staining and α-smooth muscle actin immunohistochemistry.** Mice were exposed to chlorine, and tracheas were collected for analysis 7 days after exposure. **A**. Trichrome staining of distal trachea containing fibrotic lesions. Arrows indicate collagen (blue) staining in fibrotic area. Scale bar represents 100 μm. **B**. Immunohistochemistry for α-smooth muscle actin in distal trachea showing lack of staining in an area of efficient epithelial repair. **C**. Immunohistochemistry for α-smooth muscle actin showing staining in a fibrotic region of distal trachea. Arrows indicate areas with α-smooth muscle actin within the fibrotic lesion. Arrowhead shows stronger staining in airway smooth muscle. Scale bars in panels **B** and **C** represent 20 μm. Scale bar in panel **B** represents 20 µm in **B** and **C.**

Adult male mice were exposed to a target chlorine dose of 240 ppm-hr (240 ppm chlorine for 1 hr). The effects of chlorine exposure in the trachea were initially analyzed by H&E staining (Figure 1) and quantitated by morphometric analysis (Figure 2). Preliminary studies indicated differential effects of chlorine along the proximal-distal axis of the trachea, so proximal, middle, and distal portions of the trachea were analyzed separately. In unexposed mice, examination of tracheal sections revealed a pseudostratified epithelium in proximal, middle, and distal trachea (Figure 1A-C and 2). Chlorine exposure resulted in sloughing of most if not all of the tracheal epithelium as was observed at day 2 (Figure 1D-F and 2). At this time a thin layer of squamous epithelium could be observed lining portions of the tracheal lumen (Figure 1D), whereas other areas appeared to be entirely denuded of epithelium (Figure 1E and F). Denuded areas contained eosinophilic material (possibly fibrin), inflammatory cells, and detached epithelial cells. Epithelial repair proceeded faster in the proximal trachea than in the middle or distal portions. On day 4 after exposure, many areas of proximal trachea had regenerated a normal pseudostratified epithelium (Figure 2A). Other areas showed a reparative epithelium containing undifferentiated generally cuboidal cells that tended to form multiple layers producing a pluristratified, reparative epithelium (Figure 1G and 2C). Middle and distal trachea appeared to be in earlier stages of repair, including squamous and simple cuboidal epithelia, and often still had areas devoid of epithelial cells, particularly in the distal trachea (Figure 1H and I, Figure 2). On day 7, the proximal trachea showed primarily a pseudostratified epithelium (Figure 1J), whereas and middle and distal trachea showed a mixture of pseudostratified, reparative (Figure 1K), and unrepaired (Figure 1L) epithelium (Figure 2). Repair processes appeared similar in ventrolateral trachea adjacent to cartilage and in dorsal trachea adjacent to the trachealis muscle. Areas of inefficient repair were observed mostly in distal trachea and were characterized by fibrotic lesions (Figure 1L). On day 10, distal sections showed a mixture of areas with well repaired pseudostratified epithelium (Figure 1O) along with poorly repaired areas with fibrotic lesions. Fibrotic lesions grew from the tracheal wall and protruded into the lumen to partially obstruct the airway (Figure 3). Fibrotic lesions in distal trachea occurred both ventrally over cartilage and dorsally over smooth muscle, but did appear to be more common dorsally. Similar but more pronounced occlusive fibrotic lesions were also observed in mainstem and lobar bronchi (not shown). Trichrome staining of distal tracheal confirmed the presence of collagen within fibrotic areas (Figure 4A). Fibrotic tissue also exhibited staining for α-smooth muscle actin, suggesting the presence of myofibroblasts, which are characteristic of fibrotic tissue in human pulmonary fibrosis [24,25] and in animal models of lung fibrosis [26,27].

**Figure 5:**
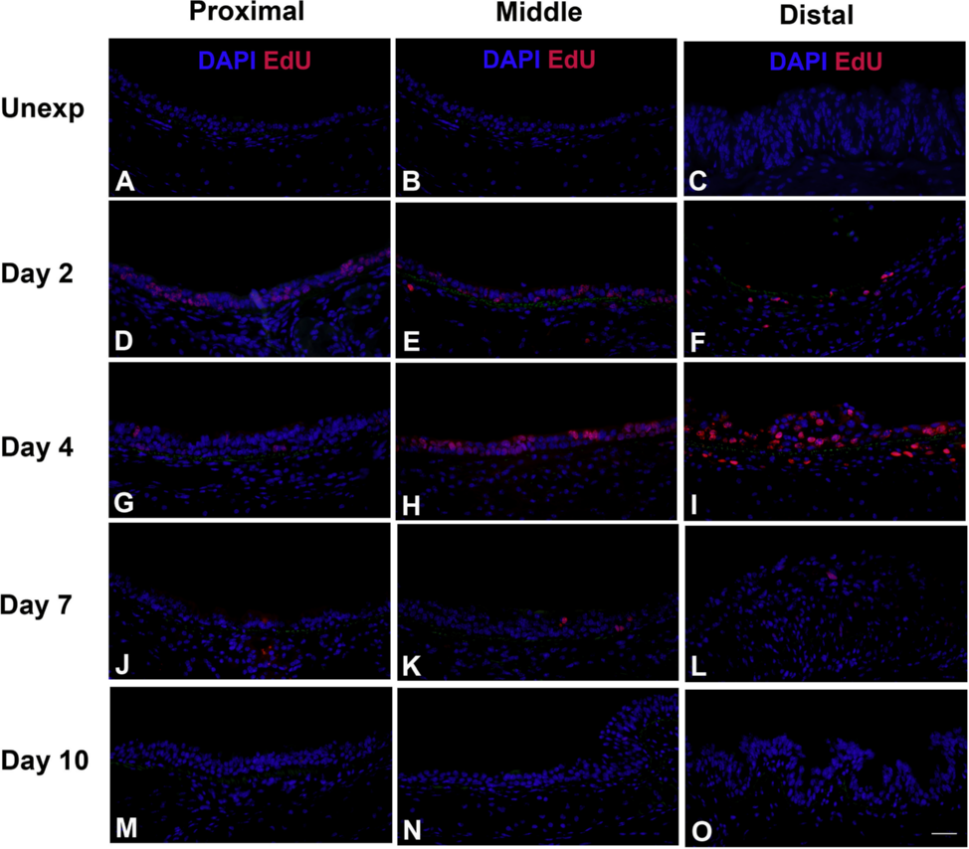
**Cell proliferation in tracheal epithelium after chlorine-induced injury.** Mice were exposed to chlorine and injected with EdU 17 hr prior to euthanasia and collection of tracheas 2, 4, 7, or 10 days after exposure. Tracheal sections were stained for EdU (red), and DAPI was used to visualize nuclei (blue). Green is tissue autofluorescence to aid in the visualization of airway structure. Scale bar in Panel **O** represents 40 μm for all panels.

**Figure 6:**
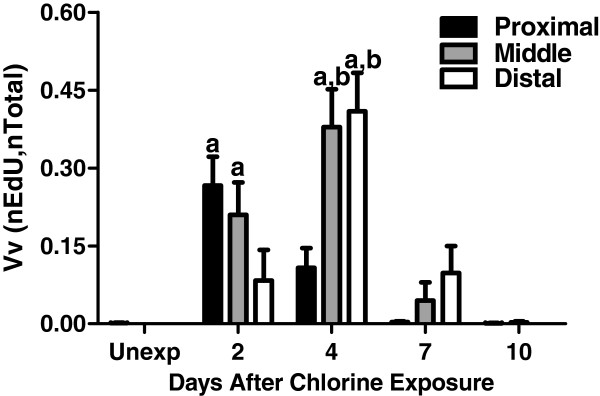
**Morphometric analysis of cell proliferation in tracheal epithelium after chlorine injury.** Cell proliferation was assessed in EdU-stained tracheal sections as described in Materials and Methods. The graphs depict the volume density (V_v_) of EdU-labeled nuclei (nEdU) relative to total nuclei in tracheal epithelium (nTotal). a, p < 0.05 vs. unexposed; b, p < 0.05 vs. proximal.

Cellular proliferation during tracheal repair after chorine exposure was detected by EdU labeling (Figure 5) and quantified by morphometry (Figure 6). Minimal proliferation was observed in unexposed mice (Figure 5A-C and Figure 6), but proliferation increased after chlorine exposure (Figure 5D-L). A peak in EdU staining was observed in the proximal trachea on day 2 (Figure 5D and Figure 6) whereas peak proliferation in the middle and distal trachea was observed on day 4 (Figure 5H and I and Figure 6). Proliferation returned to nearly normal levels by day 7 in proximal trachea and day 10 in middle and distal trachea (Figure 6).

**Figure 7:**
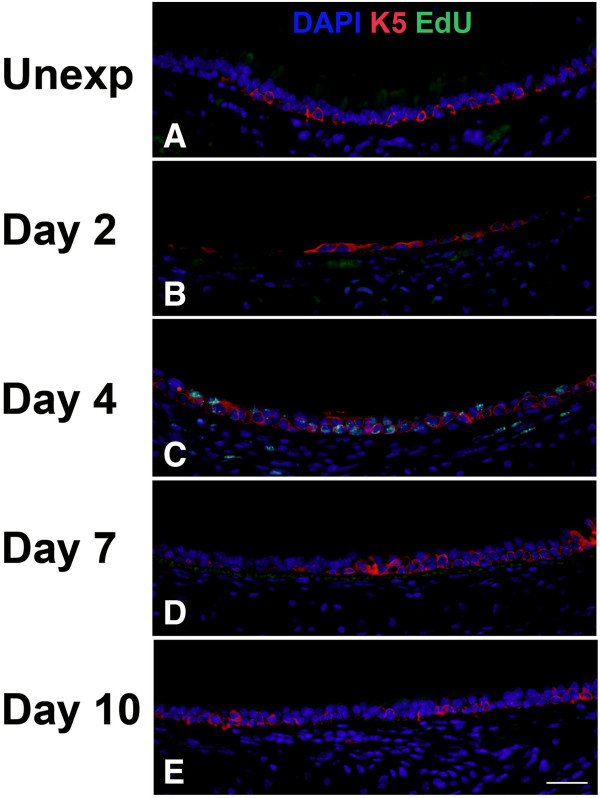
**Cell proliferation and K5 expression in middle trachea after chlorine injury.** Mice were exposed to chlorine and injected with EdU 17 hr prior to euthanasia and collection of tracheas. Tracheal sections were stained for EdU (green) and K5 (red), and nuclei were labeled with DAPI (blue). Scale bar in Panel E represents 40 μm for all panels.

**Figure 8:**
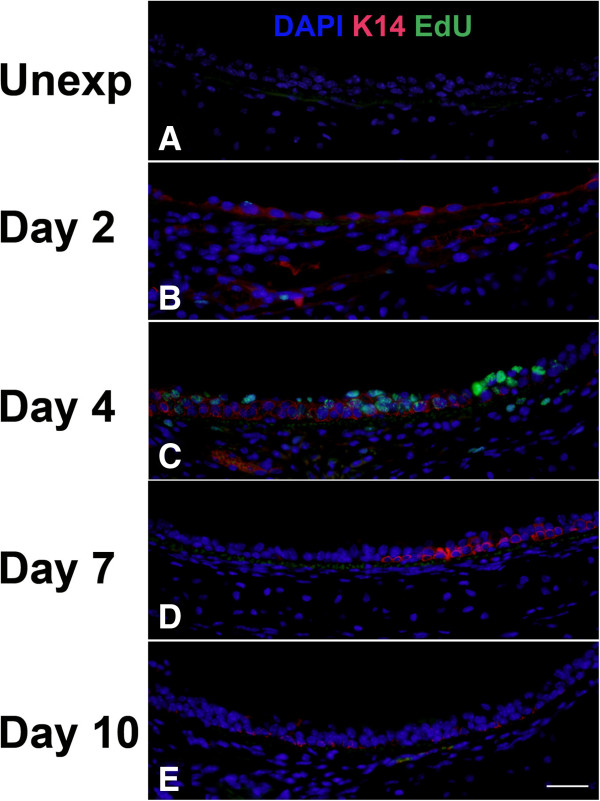
**Cell proliferation and K14 expression in middle trachea after chlorine injury.** Mice were exposed to chlorine and injected with EdU 17 hr prior to euthanasia and collection of tracheas. Tracheal sections were stained for EdU (green) and K14 (red), and nuclei were labeled with DAPI (blue). Scale bar in Panel E represents 40 μm for all panels.

**Figure 9:**
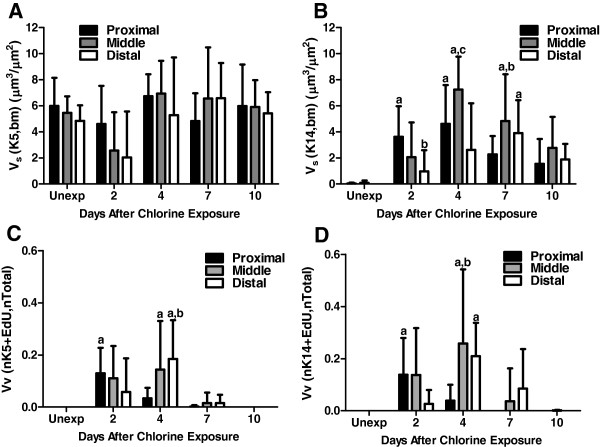
**Morphometric analysis of basal cell markers K5 and K14 in tracheal epithelium after chlorine injury.** Tracheal sections stained for EdU in conjunction with either K5 or K14 were evaluated as described in Materials and Methods. (**A**) Volume of K5-expressing cells relative to basement membrane surface area. (**B**) Volume of K14-expressing cells relative to basement membrane surface area. a, p < 0.05 vs. unexposed; b, p < 0.05 vs. proximal; c, p < 0.05 vs. distal. (**C**) Proliferation in K5 cells measured as volume density of EdU-labeled nuclei in K5-expressing cells relative to the total epithelial nuclear volume. a, p < 0.05 vs. unexposed; b, p < 0.05 vs. proximal. (**D**) Proliferation in K14 cells measured as volume density of EdU-labeled nuclei in K14-expressing cells to the total epithelial nuclear volume. a, p < 0.05 vs. unexposed; b, p < 0.05 vs. proximal.

To determine the contribution of basal cells to proliferation and repair of the tracheal epithelium, we performed EdU detection in conjunction with immunofluorescence for the basal cell markers K5 (Figure 7) or K14 (Figure 8) and quantitated the results by morphometry (Figure 9). Images of tissue staining in middle trachea are shown; similar processes occurred in proximal and distal trachea although with somewhat different kinetics. Even within the same section, it was also apparent that areas in close proximity could be at different stages of the repair process. Expression of K5 was observed in almost all basal cells in unexposed mice (Figure 7A). In contrast, there were minimal or no K14-expressing cells in unexposed mice (Figure 8A and 9B). Following chlorine exposure, both K5 and K14 staining could be seen in squamous epithelium during the early stage of repair (Figure 7B and 8B). Abundant K5- and K14-expressing cells were observed in pluristratified reparative epithelium on day 4 in both basal and luminal locations; EdU staining was common in cells expressing these markers at this stage (Figure 7C and 8C). K5 and K14 continued to be expressed at day 7, but proliferating cells were significantly reduced (Figure 7D and 8D). At this stage K5 and K14 staining was becoming more restricted to a basal location, although many of the stained cells continued to exhibit increased cellular and nuclear size compared with basal cells in unexposed mice. On day 10, K5 staining was observed predominantly in a basal location in mostly smaller cells similar to normal basal cells, although some larger cells, both in basal and luminal locations continued to be observed (Figure 7E). K14 expression was reduced compared with earlier times, but was still observed in excess of that in unexposed mice (Figure 8E). Essentially no cellular proliferation was detected at this time. Morphometric analysis revealed a nonsignificant trend toward decreased volume of K5-expressing cells at day 2 followed by recovery to normal levels by day 4 (Figure 9A, although the distribution of the staining was altered, as shown in Figure 7). The volume of K14-expressing cells increased dramatically in chlorine-exposed mice, peaking on days 2–4 and becoming lower, but still readily detectable, on day 10 (Figure 9B). An assessment of K5- and K14-expressing cells that were proliferating revealed similar kinetics with a peak in these populations at day 2 in proximal trachea and day 4 in middle and distal trachea, with proliferation returning to near zero by day 10 (Figure 9 C and D).

**Figure 10:**
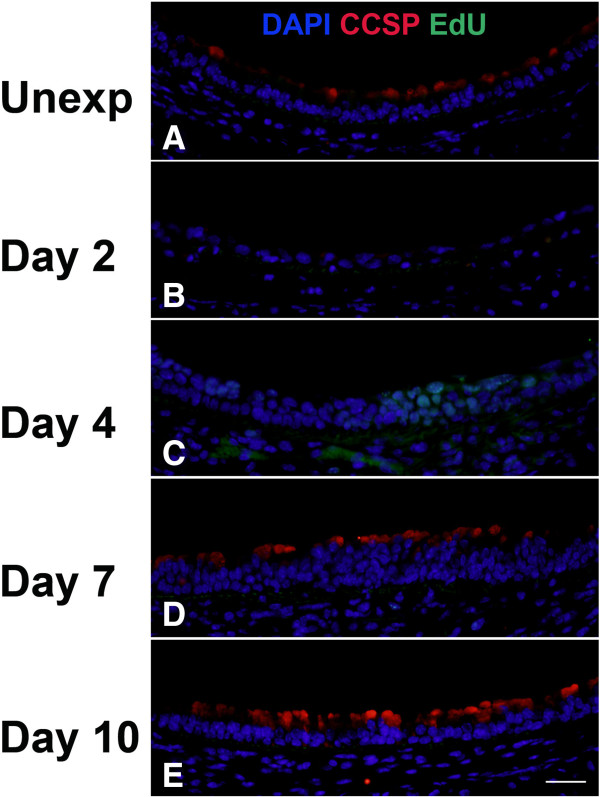
**Cell proliferation and expression of Clara cell marker CCSP in middle trachea after chlorine injury.** Mice were exposed to chlorine and injected with EdU 17 hr prior to euthanasia and collection of tracheas 2, 4, 7, and 10 days after exposure. Tracheal sections were stained for EdU (green) and CCSP (red), and nuclei were labeled with DAPI (blue). Scale bar in Panel **E** represents 40 μm for all panels.

**Figure 11:**
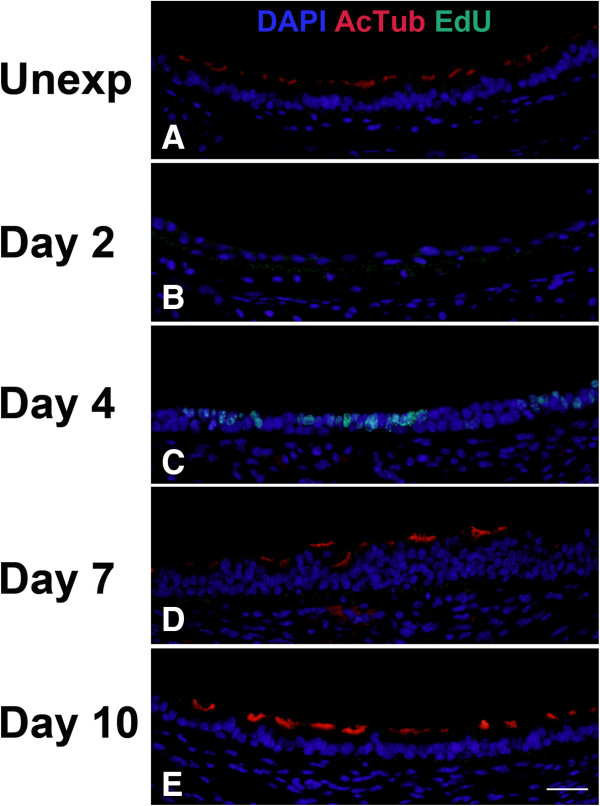
**Cell proliferation and expression of ciliated cell marker AcTub in middle trachea after chlorine injury.** Mice were exposed to chlorine and injected with EdU 17 hr prior to euthanasia and collection of tracheas 2, 4, 7, and 10 days after exposure. Tracheal sections were stained for EdU (green) and AcTub (red), and nuclei were labeled with DAPI (blue). Scale bar in Panel **E** represents 40 μm for all panels.

**Figure 12:**
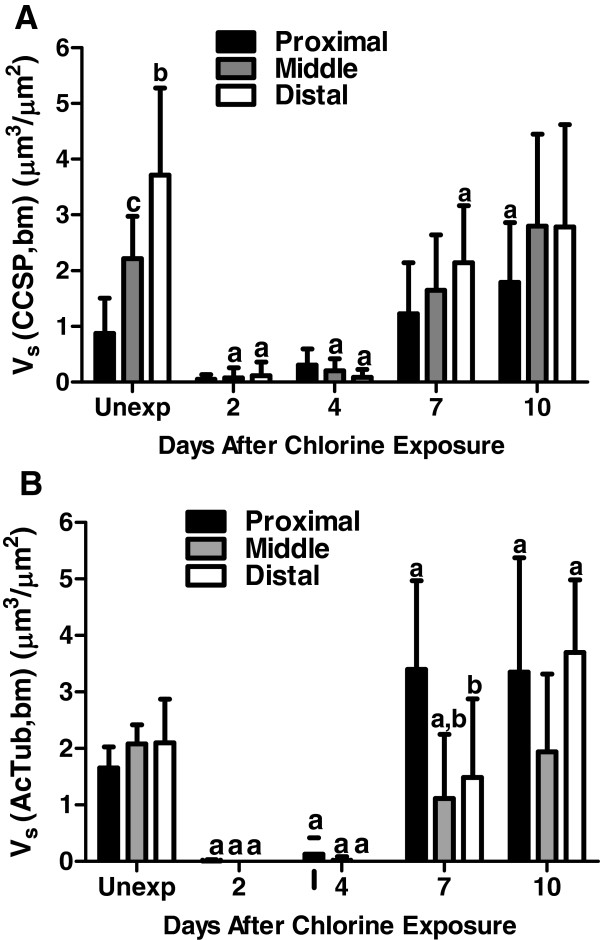
**Morphometric analysis of Clara cell and ciliated cell markers in tracheal epithelium after chlorine injury.** Tracheal sections stained for EdU in conjunction with either CCSP or AcTub were evaluated as described in Materials and Methods. (**A**) Volume of CCSP staining relative to basement membrane surface area. a, p < 0.05 vs. unexposed; b, p < 0.05 vs. proximal; c, p < 0.05 vs. distal. (**B**) Volume of AcTub staining relative to basement membrane surface area. a, p < 0.05 vs. unexposed; b, p < 0.05 vs. proximal.

EdU detection in conjunction with immunofluorescence for either Clara cell (CCSP, Figure 10) or ciliated cell (AcTub, Figure 11) markers was performed and quantitated by morphometric analysis (Figure 12). CCSP expression was detected in unexposed mice (Figure 10A), and was more abundant in the distal trachea compared with the proximal and middle trachea (Figure 12A). Chlorine exposure led to the loss of virtually all CCSP-expressing cells from the trachea (Figure 10B and 12A). During stages when proliferation was high (days 2–4), minimal if any CCSP-expressing cells were detected (Figure 10C and 12A). Expression of CCSP was observed at later times during repair at days 7 and 10 (Figure 10D, 10E, and 12A), but CCSP expression was not observed in any areas of the epithelium where there were proliferating cells. Expression of the ciliated cell marker AcTub appeared to be evenly distributed throughout the trachea in unexposed mice (Figure 11A and 12B). Chlorine exposure resulted in the loss of ciliated cells from the tracheal epithelium, and virtually no ciliated cells were observed on days 2 and 4 after chlorine exposure (Figure 11B, 11C, and 12B). Ciliated cells were observed at later times during repair (days 7 and 10) but no proliferating cells were seen in areas of the trachea where ciliated cells had differentiated (Figure 11D and 11E). On day 10, the volume of AcTub staining was increased in the proximal and distal trachea of chlorine-exposed mice compared with unexposed (Figure 12B).

## Discussion

The results of the current study define the nature and time course of epithelial repair following chlorine injury of the trachea. Exposure of mice to chlorine led to the loss of most of the tracheal epithelium, including virtually all Clara and ciliated cells. During steady state, minimal cell proliferation was detected; however after chlorine injury, an upsurge in cell proliferation was observed. The proximal trachea had an earlier initiation of repair and faster restoration of pseudostratified epithelium than did the distal trachea. During repair, numerous proliferating K5- and K14-expressing basal cells were detected. In contrast, Clara and ciliated cells were absent during the proliferative stage of repair, and markers for these differentiated cell types were only observed at later time points. Fibroproliferative lesions developed in areas where epithelial repair was inefficient.

The results presented here provide evidence that basal cells function as progenitor cells in the initiation of repair in the tracheal epithelium after chlorine injury. With the loss of most epithelial cells after chlorine injury, basal cells were the only surviving cells initially observed in the tracheal lumen as a thin layer of squamous epithelium that served to repopulate the trachea. The abundant and widespread distribution of basal cells across the tracheal epithelium during the repair process is more consistent with basal cells functioning as progenitor cells rather than as a rare population of stem cells. Prior studies have demonstrated that basal cells of the tracheobronchial epithelium constitute a multipotent progenitor cell population capable of self-renewal and of differentiation into Clara and ciliated cells after injury [11,12,14,15,18]. Our results are consistent with basal cells being the major progenitor cell effecting repair after chlorine injury. Clara cells have also been identified as a cell population capable of self-renewal that can contribute to the restoration of injured airway epithelium [16,17], including the trachea [28]. In the present study, however, few if any Clara cells were detected during the peak period of cell proliferation, and no EdU-labeled Clara cells were observed after chlorine lung injury. Following sulfur dioxide injury, basal cells were concluded to be the major progenitor cells involved in repair, with Clara cells that survived the injury playing a minor role in repopulating the trachea with Clara and ciliated cells [12,28]. We exposed mice to chlorine doses high enough to kill virtually all the Clara cells of the trachea, so the Clara cells observed starting 7 days after exposure appear to be derived from basal cells that survive chlorine exposure. As we did not detect proliferation in these regenerated Clara cells, they do not appear to play any major role as progenitor cells for tracheal repair following a high dose of chlorine. This is clearly different from the case of naphthalene injury in the bronchiolar epithelium, in which repair is carried out primarily by surviving Clara cells, and the majority of proliferating epithelial cells after injury are CCSP-positive. However, our results do not rule out the possibility that Clara cells may participate in tracheal repair following exposure to lower doses of chlorine that allow greater survival of these cells.

Following chlorine injury we identified a dynamic pattern in the expression of cytokeratins that have been used as basal cell markers. During normal steady state, most basal cells express K5 but a smaller subset expresses K14 [11,14,15] as we observed in this study. The increase we observed in K14-expressing cells during repair is similar to findings reported after naphthalene injury [14,15,18]. In other tissues such as skin and cornea, K5 and K14 are typically co-expressed and represent binding partners in heterodimeric keratins [29]. The binding partner for K5 in normal mouse tracheal epithelium appears to be keratin 15 [11,15]. The upregulation of K14 following injury and its association with the proliferative stage suggests a specific function for this molecule in the repair of the tracheobronchial epithelium. We observed drastic changes in the size and shape of K5- and K14-expressing cells during the course of repair, progressing from thin cells covering the basement membrane to larger cells with enlarged nuclei in the pluristratified reparative epithelium and then returning to small pyramidal cells coincident with loss of K14 expression. As keratins are involved in controlling cell shape and motility, the transient expression of K14 may play a role in the dynamic changes in epithelial morphology that occur during the repair process.

Repair of the tracheal epithelium varied along the proximal-distal axis, with epithelial restoration occurring faster and more completely in the proximal trachea than in the distal trachea or mainstem bronchus. A possible explanation for these observations is that the proximal trachea possesses better intrinsic repair capacity as opposed to the middle and distal trachea or mainstem bronchus. An alternative possibility, which we consider more likely, is that chlorine inhalation results in less initial damage to the epithelium of the proximal trachea. This concept is supported by the observation that initial injury at day 2 after chlorine exposure appeared to be more severe in distal trachea as revealed by an increased percentage of denuded airway surface area. Submucosal glands, which are restricted to the proximal trachea in mice, have been suggested to serve as a protective niche for basal stem cells [10]. The abundance and widespread distribution of basal cell progenitors involved in the repair process that we observed do not support the migration of basal cells from localized stem cell niches as a major contributor in the restoration of the tracheal epithelium. In distal trachea, we observed a trend toward decreased K5 staining suggestive of fewer basal cells (which could result in less efficient repair), but the difference was not statistically significant. It is possible that other differences in anatomy may result in differential protection of the proximal airway epithelium, leading to fewer denuded areas and more efficient repair.

The initial regeneration of the pseudostratified epithelium in well-defined areas occurred within 7 days after chlorine exposure, but it was apparent that the epithelium at this point was not yet repaired to its original state. At 10 days after exposure, even within well repaired areas, significant differences between regenerated and unexposed epithelium could be observed, including increased expression of K14 in basal cells and increased ciliated cells. We did not determine whether these parameters returned to normal within well repaired areas at later times because of the death of a significant number of animals that occurred between days 7 and 14. Delayed lethality 7–14 days after exposure has been observed previously following inhalation of chlorine in mice [30]. In our experiments, lethality appeared to be associated with airway fibrosis and tracheal or bronchial stenosis in areas of inefficient epithelial repair. Chlorine exposure in dogs resulted in chronic effects of bronchiolitis obliterans, which appeared to be a similar process to what we observed in mice except that it occurred in smaller airways [31]. In mice, a pseudostratified epithelium with basal cells is restricted to the tracheobronchial region, whereas in larger mammals, including humans, basal cells extend farther into the lung to the level of smaller bronchioles [10]. The more extensive distribution of basal cells in larger mammals may provide for better repair of epithelium in the proximal airways, allowing survival of animals following extensive desquamation of the tracheobronchial epithelium, as was observed in dogs [31].

Previous studies have examined the repair of airway epithelium after chlorine exposure in mice [6] and rats [32]. These studies focused on intrapulmonary airways in contrast to the processes occurring in the trachea that were investigated in the present study. The rodent tracheal epithelium has been proposed as a model of human airway epithelium, which contains a pseudostratified epithelium with abundant basal cells that extends to the smaller airways [10]. Exposure of A/J mice to 800 ppm chlorine for 5 min resulted in death and sloughing of epithelium from intrapulmonary airways, stimulation of cellular proliferation, and airway remodeling characterized by increased smooth muscle and collagen [6]. The time course of repair appeared similar to what was observed in the trachea, with a peak in cellular proliferation 5 days after exposure and restoration of a normal epithelium by 10 days. The cells types carrying out repair in the intrapulmonary airways were not investigated, but may possibly be basal cells in the larger airways and Clara cells in the bronchioles by analogy to other injury models (discussed below). Exposure of rats to 400 ppm chlorine for 30 min resulted in sloughing of epithelium from large intrapulmonary airways [32]. The epithelium was repaired by day 7 after exposure, but was abnormal in that it was thickened and contained increased mucus-producing cells. The repaired tracheal epithelium also showed abnormalities, including altered distribution of ciliated cells and increased K14 staining. Thus a common result between the studies is that repair occurs rapidly, but, at least initially, does not restore the epithelium to its normal state. Further studies are required to assess whether any of these epithelial abnormalities resolve over longer time periods.

Our observations are consistent with a model in which surviving basal cells function as progenitor cells to repopulate the tracheal epithelium after chlorine injury. Repair of the airway epithelium has been previously studied using naphthalene [18,28,33-35] and sulfur dioxide [12,28,36]. In the tracheal epithelium, naphthalene injury to Clara cells results in depletion of both Clara cells and ciliated cells, and repair is initiated by a hyperplasia of K5 and K14-labeled basal cells that serve as progenitors for Clara and ciliated cells [11]. In this case repair is efficient because, although Clara and ciliated cells are lost, the full complement of basal cells remains. In contrast, sulfur dioxide, like chlorine, has the potential to injure all epithelial cell types, and the dose of sulfur dioxide used in previous studies resulted in extensive sloughing of tracheal epithelial cells [12,37]. Repair of the tracheal epithelium was found to be carried out primarily by basal cells but also by Clara cells that survived sulfur dioxide exposure [12,28]. The dose of chlorine used in our studies appears to result in more severe injury, as large areas of the trachea were either denuded or lined with squamous epithelium, rather than a cuboidal monolayer as observed following sulfur dioxide inhalation [28,37]. This suggests that significant numbers of basal cells are lost following high-dose chlorine exposure and this limits or delays repair. Despite the differences in mechanism and extent of epithelial damage, the airway injury models reveal a general response to tracheal epithelial loss that involves the migration, proliferation, and differentiation of basal cells to restore the integrity of the epithelium. Understanding these concepts in the context of chlorine lung injury provides the potential opportunity to manipulate the repair process to accelerate normal epithelial healing following exposure to this chemical threat agent.

## Conclusions

Chlorine inhalation resulted in the death and sloughing of virtually all Clara and ciliated cells and a variable number of basal cells from the trachea. Surviving basal cells spread to cover the injured airway and proliferated to restore a pseudostratified epithelium containing Clara and ciliated cells. In areas with few surviving basal cells, repair was inefficient, leading to an extended period of denuded basement membrane and the development of airway fibrosis. These studies establish a model for understanding regenerative processes in the respiratory epithelium that will be useful for testing therapies for airway injury based on stimulation of epithelial repair.

## Abbreviations

AcTub: Acetylated tubulin; ANOVA: Analysis of variance; CCSP: Clara cell secretory protein; EdU: 5-ethynyl-2^′^-deoxyuridine; K5: Keratin 5; K14: Keratin 14; Unexp: Unexposed.

## Competing interests

The authors declare that they have no competing interests.

## Authors’ contributions

SM and GWH conceived and designed the experiments; SM and JC performed the experiments; SM and GWH analyzed data and interpreted results of experiments; SM and GWH prepared figures; SM drafted the manuscript; SM and GWH edited and revised the manuscript; SM, JC, and GWH approved the final version of the manuscript.
